# Phylogeography of the Chinese Beard Eel, *Cirrhimuraena chinensis* Kaup, Inferred from Mitochondrial DNA: A Range Expansion after the Last Glacial Maximum

**DOI:** 10.3390/ijms150813564

**Published:** 2014-08-05

**Authors:** Hai Li, Hungdu Lin, Jianlong Li, Shaoxiong Ding

**Affiliations:** 1 State Key Laboratory of Marine Environmental Science, Xiamen University, Xiamen 361102, China; E-Mails: josephlee@stu.xmu.edu.cn (H.L.); haikuilee@gmail.com (J.L.); 2 The Laboratory of Marine Biodiversity and Global Change, Xiamen University, Xiamen 361102, China; 3 Department of Physical Therapy, Shu Zen College of Medicine and Management, Kaohsiung 821, Taiwan; E-Mail: varicorhinus@hotmail.com

**Keywords:** phylogeography, *Cirrhimuraena chinensis* Kaup, population expansion, last glacial maximum, larval dispersal

## Abstract

The Chinese beard eel (*Cirrhimuraena chinensis* Kaup) is an intertidal fish and a model organism for the study of impacts caused by topological fluctuations during the Pleistocene and current intricate hydrological conditions on fauna living in the coastal areas of China. In this study, we examined the phylogeographical pattern, population genetic profile and demographical history of *C. chinensis* using mitochondrial DNA (cytochrome *b* (cyt *b*) and control region (CR)) from 266 individuals sampled in seven localities across the coastal area of southeastern China. The combined data indicated high levels of haplotype diversity and low levels of nucleotide diversity. Analyses of molecular variance (AMOVA) and *F*_ST_ statistics suggested the absence of a significant population structure across the Chinese coast. Neutrality tests, mismatch distributions and Bayesian skyline plots uniformly indicated a recent population expansion. The phylogeographic structure of *C. chinensis* may be attributed to past population expansion and long-distance pelagic larval dispersal facilitated by present-day ocean currents.

## 1. Introduction

Characterized by a series of periodic climate oscillations, the Quaternary period is thought to have had profound influences on the development of many flora and fauna in both terrestrial and aquatic environments [1,2]. The fall and rise of sea levels caused by the advancing and retreating of ice sheets during the Pleistocene are expected to have produced conspicuous genetic effects affecting intraspecific differentiation and population structure in a number of marine species through alteration of their geographic range and abundance on both spatial and temporal scales [2,3]. Such events could have a significant impact for organisms distributed in marginal sea areas, where ocean basins were intermittently isolated and connected during glacial-interglacial cycles. Containing 75% of the world’s marginal sea area [4], the Northwestern Pacific area has undergone severe transformation during ice ages. For example, during the last glacial maximum (LGM), the sea level dropped to 130~150 m below and 100~120 m below the present level in the East China Sea (ECS) and South China Sea (SCS), respectively [5], which caused extensive habitat loss for many marine species and the fragmentation of their populations by newly emerged dispersal barriers. Former surveys have demonstrated that paleoclimatic fluctuations, along with contemporary oceanic factors, can play a significant role in shaping the evolutionary history of species living in coastal areas [6,7,8]. In this context, the phylogeographic study of marine organisms inhabiting the Northwestern Pacific area has unique implications for interpreting the effects that historical events, geological configurations and contemporary oceanographic features have on the biogeographic distribution and the biodiversity of marine species [9].

Intertidal organisms form a biotic assemblage occupying a narrow, discrete and vulnerable strip of closely linked land and sea [10] and are consistently associated with dynamic variations of the current oceanographic regime. On the one hand, they are inclined to exhibit phylogeographic heterogeneity, as abrupt discontinuities between adjacent populations resulting from vicariance events may give rise to reciprocal monophyly between populations [11]; on the other hand, various intertidal species have a planktonic larval phase that enables them to disperse for days or weeks before settling [12,13,14]; and because pelagic larval duration (PLD), which is closely related to regional hydrographic conditions and ecological factors, varies among species, the level of gene flow that contributes toward genetic homogeneity among separated biomes remains uncertain. Therefore, intertidal organisms are believed to be excellent models to shed light on the genetic variation and differentiation comprehensively impacted by the potential historical and current factors [10]. However, to date, studies aiming to elucidate the phylogeographic patterns of intertidal organisms in the Northwestern Pacific area, particularly in the coastal areas of China, have mainly focused on invertebrates, such as the limpet, *Cellana toreuma* [8], the cold-water barnacle, *Chthamalus challengeri* [15], and the bivalve, *Cyclina sinensis* [16]. Relevant studies on intertidal fishes have yet to be reported on a wide geographic scale along the coast of China.

The Chinese beard eel (*Cirrhimuraena chinensis* Kaup) is a nearshore warm-water demersal fish, distributed from the eastern coast of Indo-Africa to Indonesia, China and the Philippines. In China, it occurs along the coastal areas of the East China Sea, Taiwan Strait and South China Sea [17]. It inhabits sandy shallows of estuaries and burrows of low intertidal zones and is generally considered to be a resident fish without a long-distance swimming ability [18]. In addition, its lifecycle contains a leptocephalic stage [19], as do those of most Anguilliformes fishes, which implies the existence of a planktonic larval phase. The discrepancy between the dispersal ability of *C. chinensis* larvae and adults therefore makes this species a good model organism to test the complex impacts brought about by sea level change during the Pleistocene and by current intricate hydrological conditions on fauna living in the coastal areas of China.

In the present study, we examined the phylogeography of *C. chinensis* using two mitochondrial DNA (mtDNA) sequences, cytochrome *b* (cyt *b*) and the control region (CR), with the aim of revealing the genetic profile of the *C. chinensis* population and its demographic history. By addressing these issues, we expect to provide further insights into the evolutionary mechanisms that shape the phylogeography of marine fauna living in the coastal areas of China and to elucidate the influences that medium to long pelagic larval stages have on the phylogeographic pattern for intertidal fishes.

## 2. Results

### 2.1. Genetic Diversity

A dataset was obtained comprising 266 individuals and containing two sequenced fragments, one 804-bp fragment from the cytochrome *b* coding sequence and one 836-bp fragment from the D-loop. A total of 131 variable sites were observed, of which 87 were parsimony-informative and 160 haplotypes were observed. The overall haplotype diversity (*h*) and nucleotide diversity (π) were 0.967 and 0.002, respectively. Detailed information about haplotype, haplotype diversity and nucleotide diversity for each sampling locality is shown in Table 1.

**Table 1:** Summary of sample size, haplotype numbers, haplotype diversity (*h*), nucleotide diversity (π), Tajima’s D, Fu’s *F*s, Ramos–Onsins & Rozas’ *R*_2_ tests and goodness-of-fit tests for cytochrome *b* (cyt *b*), the control region (CR) and cyt *b* + CR sequences in each population, respectively. SSD, sum of square deviation. * *p* < 0.05; and ** *p* < 0.01.

Populations (Abbreviation)	Sample Size (*n*)	Haplotype Numbers	Haplotype Diversity (*h*)	Nucleotide Diversity (π)	Tajima’s *D*	Fu’s *F*s	Ramos–Onsins & Rozas’ *R*_2_	SSD	Raggedness Index
**cyt *b***	266	64	0.700	0.002	−2.569 **	−28.372 **	0.010 **	0.000	0.022
**CR**	266	112	0.916	0.003	−2.340 **	−26.782 **	0.010 **	0.001	0.044
**Ningde(ND)**	49	45	0.997	0.003	−2.164 **	−25.812 **	0.032 **	0.008	0.033
**Xiamen(XM)**	37	24	0.943	0.002	−2.663 **	−20.242 **	0.035 **	0.001	0.020
**Shantou(ST)**	30	27	0.991	0.002	−2.062 **	−25.791 **	0.043 **	0.010	0.041
**Yangjiang(YJ)**	50	36	0.964	0.002	−2.521 **	−26.174 **	0.024 **	0.001	0.025
**Beihai(BH)**	35	28	0.978	0.003	−1.932 **	−25.372 **	0.044 **	0.001	0.016
**Haikou(HK)**	37	24	0.929	0.002	−1.893 **	−18.808 **	0.044 **	0.104	0.020
**Sanya(SY)**	28	7	0.915	0.002	−1.808 *	−12.332 **	0.057 **	0.098	0.026
**Total**	266	160	0.967	0.002	−2.538 **	−25.652 **	0.012 **	0.001	0.017

### 2.2. Population Structure Pattern

Pairwise *F*_ST_ values were low and ranged from −0.007 to 0.005, with an average value of 0.001 (Table 2). Analyses of molecular variance (AMOVA) of all scenarios consistently revealed that most of the variations in concatenated sequences (>99%) were attributable to genetic variations within populations, while only a small amount of the variations (<1%) in concatenated sequences was associated with variations between populations or between groups (Table 3). For the hierarchical analysis, groupings based on several putative geological barriers all exhibited low *F*_CT_ values (Table 3). A Mantel test (*r* = 0.01396, *p* < 0.05) did not detect any correlation between genetic distance and geographic distance for concatenated sequence data.

**Table 2:** Matrix of pairwise of *F*_ST_ across seven populations based on cyt *b* + CR sequences in *Cirrhimuraena chinensis*.

cyt *b* + CR	ND	XM	ST	YJ	BH	HK	SY
**ND**							
**XM**	0.00216						
**ST**	−0.00717	−0.00612					
**YJ**	0.00359	−0.00241	0.00158				
**BH**	−0.00492	−0.00350	−0.01347	0.00157			
**HK**	0.00754	0.00423	−0.00378	0.00535	0.00391		
**SY**	0.00407	0.00458	−0.00086	0.00663	0.01164	0.01176	

*p* values of *F*_ST_ (not presented here) are all greater than 0.05 and 0.01, which means all the *F*_ST_ values in the table above are not statistically significant under *p* < 0.05 and *p* < 0.01)

**Table 3:** Analyses of molecular variance (AMOVA) results for testing the genetic subdivision of populations using cyt *b* + CR sequences across geographic districts.

cyt *b* + CR	Sum of Squares	Percentage of Variation	Fixation Indices	Significance Tests
Groups: Taiwan strait (ND) (XM, ST, YJ, BH, HK, SY)				
Among groups	2.178	0.18	Ф_CT_ = 0.00182	*p* = 0.580
Among populations within groups	9.447	0.11	Ф_SC_ = 0.00125	*p* = 0.264
Within populations	469.925	99.70	Ф_ST_ = 0.00297	*p* = 0.277
Groups: Pearl River (ND, XM, ST) (YJ, BH, HK, SY)				
Among groups	1.463	−0.28	Ф_CT_ = −0.00283	*p* = 0.906
Among populations within groups	10.161	0.33	Ф_SC_ = 0.00324	*p* = 0.130
Within populations	469.925	99.96	Ф_ST_ = 0.00042	*p* = 0.236
Groups: Leizhou Peninsula (ND, XM, ST, YJ, HK, SY) (BH)				
Among groups	2.010	0.09	Ф_CT_ = 0.00085	*p* = 0.390
Among populations within groups	9.614	0.16	Ф_SC_ = 0.00156	*p* = 0.223
Within populations	469.925	99.75	Ф_ST_ = 0.00242	*p* = 0.213
Groups: Qiongzhou Strait (ND, XM, ST, YJ, BH) (HK, SY)				
Among groups	2.170	0.16	Ф_CT_ = 0.00159	*p* = 0.246
Among populations within groups	9.454	0.11	Ф_SC_ = 0.00110	*p* = 0.359
Within populations	469.925	99.73	Ф_ST_ = 0.00269	*p* = 0.247

### 2.3. Historical Demography

**Figure 1:**
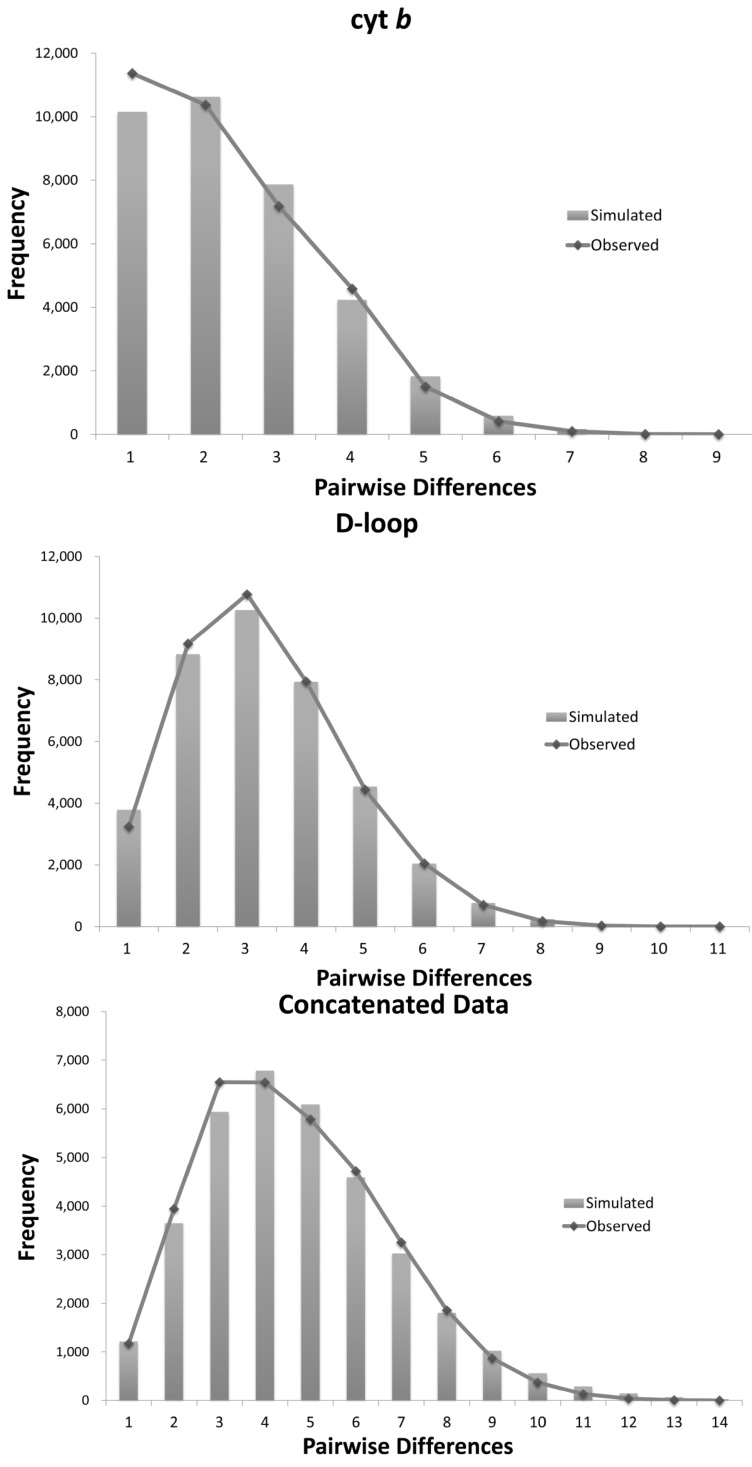
Mismatch distribution analyses of *Cirrhimuraena chinensis* Kaup for cyt *b*, CR and concatenated sequences (cyt *b* + CR).

**Figure 2:**
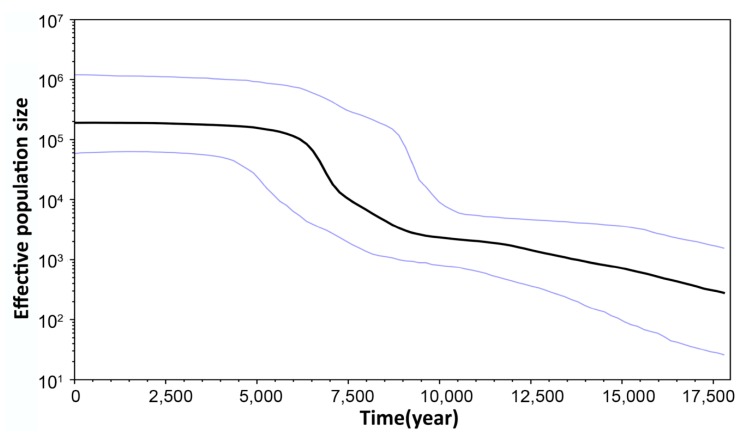
Bayesian skyline plots of the effective sizes through time for *Cirrhimuraena chinensis* Kaup based on concatenated sequences (cyt *b* + CR). The upper and lower limits of the light blue trend represent the 95% confidence intervals of highest posterior densities (HPD) analysis.

Neutrality tests of Tajima’s *D* and Fu’s *F*_S_ all yielded significant negative values, while the results of tests of Ramos–Onsins and Rozas’ *R*_2_ were all statistically significant (Table 1). Unimodal distributions were observed for the total population (Figure 1) The SSD ranged from 0.0006 to 0.1044 and was not statistically significant, while Harpending’s raggedness index displayed low values (Table 1). These results support a precise fit between the observed distribution and the expected distribution. The time since expansion was estimated to be 15,900 years ago. The Bayesian skyline plots revealed a detailed demographic history of population size changes, from which we could see a low effective population size during the Quaternary period that slowly increased from 17,500 years ago, but that increased sharply after the LGM approximately 7500 years before the present (Figure 2).

## 3. Discussion

### 3.1. Population Genetic Structure and Phylogeographic Pattern

No significant population structure was observed along a large stretch of Chinese coast within the range of *C. chinensis*. There was no evident genetic differentiation corresponding to any of the geographical partitions that were assumed to have possibly impeded genetic exchange. The absence of significant genetic differentiation and a non-significant correlation between *F*_ST_ and geographical distances suggested that a high level of genetic exchange existed between all the sampling localities. The robust genetic connectivity that shaped the genetic architecture indicated that *C. chinensis* was capable of dispersing over quite a large distance along the coastline.

Marine organisms with high dispersal capacity generally show low levels of genetic differentiation, even between distant regions [2,20,21], where physical barriers between ocean basins or adjacent continental margins are absent. A growing number of surveys have revealed the absence of significant population structure in various marine organisms with high dispersal potential, including species with excellent swimming ability, such as bigeye tuna (*Thunnus obesus*) [22], Japanese anchovy (*Engraulis japonicus*) [23] and yellow croaker (*Nibea albiflora*) [24], and some other marine species that demonstrate effective long-distance larval dispersal, such as the mud crab (*Scylla paramamosain*) [23], the cold-water barnacle (*Chthamalus challengeri*) [15], the shanny (*Lipophrys pholis*) [25] and the Japanese conger eel (*Conger myriaster*) [26]. The planktonic larval period of a number of marine species plays a crucial role in their ecology and evolution [27,28]. The life history of many marine organisms involves a pelagic larval stage, which may enable them to disperse over considerably large geographical distances and may thus yield high genetic connectivity. *C. chinensis* is usually treated as a resident fish without a long-distance swimming ability [18], yet a previous study had confirmed that *C. chinensis* undergoes a leptocephalic stage lasting 20 to 25 days at 18 to 28 °C before metamorphosing into a juvenile fish [19], which suggested that *C. chinensis* larvae are capable of dispersing for several weeks under natural oceanic circumstances. The absence of genetic structure in this species may be reasonably explained by the planktonic larval stage, playing a critical part in facilitating gene flow.

Near China’s coasts, the ocean current pattern is always intricate and variable. Water exchange and seasonal reversals in surface oceanic circulation are driven by a southwest summer and northeast winter current driven by prevailing monsoonal winds [29,30,31]. Although the velocity of the China Coastal Current varies greatly, an average value of 20 cm/s is quite common in the studied area [32]. Because the dispersal distance of many marine organisms is directly related to the time they spend in the planktonic larval stage [14], *C. chinensis* was speculated to travel a long range, considering the length of its larval stage and the current velocity in the East China Sea, South China Sea and Taiwan Strait. Therefore, the oceanographic conditions within the studied range provide *C. chinensis* with appropriate natural conditions to migrate along the Chinese coast and maintain high levels of genetic connectivity among geographically separated demes. In China, *C. chinensis* does not display any genetic breaks or distinct lineages, but instead shows a homogeneous phylogeographic pattern.

### 3.2. Historical Demography and Population Expansion

A hypothesis of population expansion was in accordance with the significant negative values of the neutrality tests (Tajima’s *D*, Fu’s *F*s), the results of the Ramos–Onsins and Rozas’ *R*_2_ test and the unimodal mismatch distribution curves. Additionally, the Bayesian skyline plots also provided evidence for the occurrence of a sudden population expansion approximately 7.5 thousand years (kyr) ago. Moreover, according to the four scenarios proposed by Grant and Bowen (1998) based on genetic diversity and nucleotide diversity, populations of *C. chinensis* with a high *h* value and a low π value most likely underwent a population expansion after a long-term period of low effective population size. All of these results therefore consistently support the hypothesis that *C. chinensis* populations along the Chinese coastal areas have experienced a population expansion.

The late Pleistocene period (the past one million years) was dominated by a series of large glacial-interglacial oscillations [33], characterized by glacial cycles occurring at ~100 kyr intervals over the past ~800 kyr [34]. Environmental change in the Pleistocene not only influenced population expansions and contractions, but also altered demographic dynamics. During the last glacial period (approximately 12~75 kyr ago), sea level was drastically lowered in the marginal seas of China (100~120 m in the South China Sea and 130~150 m in the East China Sea) [35]. The regression of glacial sea level caused the East China Sea to reduce to an elongated trough, the Okinawa Trough, and turned the South China Sea into a semi-closed inland sea connected to the Pacific through the Bashi Strait between Taiwan and Luzon [5].

The following scenario offers a plausible explanation for the post-LGM demographic expansion of *C. chinensis* detected in our study. Low sea level led to an expansion in the spectrum of salinity and temperature in nearshore waters. Deleterious niche stresses, coupled with a reduction in formerly available habitat, would have caused massive extinction and a significant reduction in population size for numerous neritic species [9]. Being an intertidal species, *C. chinensis* was closely affected by both terrestrial and marine factors; hence, it most likely experienced more severe environmental stresses than oceanic organisms [36]. Therefore, the alterations in topological configurations that occurred in the East China Sea and the South China Sea would have caused intensive coastal habitat loss and a great decrease in population size for *C. chinensis*. The remaining individuals could only have survived in potential refugia (such as the Okinawa Trough or semi-closed South China Sea) during the glacial period. With the rise of sea level after the LGM, surviving *C. chinensis* would have recolonized from the refugia and reoccupied the newly available habitats quickly [37]. As pelagic larval *C. chinensis* could disperse over a considerable distance by taking advantage of surface current circulations, its geographic distribution range would have been broadened, and its abundance and effective population size would have increased.

## 4. Materials and Methods

### 4.1. Sample Collection

**Figure 3:**
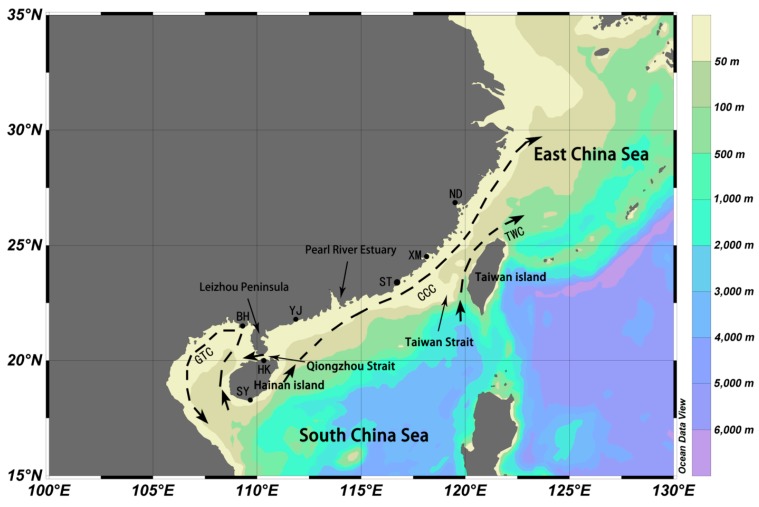
Map showing the seven sampling sites along the Chinese coast. CCC, China Coastal Current; TWC, Taiwan Warm Current; and GTC, Gulf of Tonkin Current.

Two hundred and sixty six individuals of *C. chinensis* were collected from December 2010, to May 2012, from seven localities along the coast of China, including the East China Sea, Taiwan Strait and South China Sea (Figure 3), covering the entire distribution range of *C. chinensis* in China. All of the samples were originally preserved in absolute ethanol and then refrigerated at −20 °C before DNA extraction.

### 4.2. DNA Extraction, Amplification and Sequencing

Genomic DNA was isolated from muscle tissue by proteinase K digestion using standard phenol-chloroform methods [38]. The cyt *b* fragments and CR fragments were amplified by F30 (5'-TTCGTAATACTCACGCCAACG-3') and R916 (5'-TTTCTACTCAACCCGCTAATG-3') and by F59 (5'-TCAAAGGAATTAAATGGCG-3') and R973 (5'-GTGGACAGGGACAAGGGTA-3'), respectively. The PCR amplification was carried out in a 25-μL reaction volume containing 10 mM Tris-HCl (pH 8.3), 50 mM KCl, 1.5 mM MgCl_2_, 0.2 mM of each dNTP, 0.4 μM each primer, 1 U *Taq* polymerase (TAKARA, Otsu, Japan) and 30 ng template DNA. For cyt *b* fragments, the PCR procedure consisted of an initial denaturation step at 94 °C for 3 min, followed by 30 cycles of denaturation at 94 °C for 45 s, 52 °C for 1 min, extension at 72 °C for 1 min and a final extension at 72 °C for 10 min. For CR fragments, the PCR profile consisted of an initial denaturation step at 94 °C for 3 min, followed by 30 cycles of denaturation at 94 °C for 30 s, 55 °C for 45 s, extension at 72 °C for 1 min and a final extension at 72 °C for 10 min. The newly isolated nucleotide sequences were deposited in GenBank under Accession Numbers KJ150737-KJ151281.

### 4.3. Data Analysis

cyt *b* and CR sequences were edited and aligned in Sequencher version 4.1.4 (Gene Codes Corporation, Ann Arbor, MI, USA) before being incorporated into the concatenated dataset. Genetic diversity estimates of cyt *b*, CR sequences and the concatenated dataset, including the haplotype diversity (*h*) and nucleotide diversity (π), were calculated for each location and the entire population by DnaSP version 5.0 [39].

To evaluate the genetic differentiation between sampling locations, population genetic distances (*F*_ST_) were estimated using concatenated data by Arlequin version 3.5 [40]. Analysis of molecular variation (AMOVA) was implemented in Arlequin to investigate the partition of genetic variation within and among sampling locations, along with hierarchical analysis by population groupings to investigate the possible effects of major potential geographical barriers located in the sampling range, including the Taiwan Strait, the Pearl River, Leizhou Peninsula and the Qiongzhou Strait, on the phylogeographic pattern: (1) Ningde as a single group (ND), with the remaining populations on the other side of the Taiwan Strait constituting another group; (2) populations were divided into an eastern group (ND, XM, ST) and a western group (YJ, BH, HK, SY) at the boundary of the Pearl River; (3) Leizhou Peninsula spilt the populations into an eastern group (ND, XM, ST, YJ) and a western group (BH, HK, SY); (4) populations on Hainan island belonged to an island group (HK, SY), while the mainland group (ND, XM, ST, YJ) contained the remainder in the case that the Qiongzhou Strait served as a vicariant barrier. A Mantel test was conducted to assess the significance of the relationship between the pairwise *F*_ST_ matrix and the matrix of geographic distance.

Historical demography was inferred using three approaches. First, DnaSP was used to conduct neutrality tests of Tajima’s *D* [41], Fu’s *F*_S_ [42] and Ramos–Onsins and Rozas’ *R*_2_ [43] to detect whether there was any deviation from the assumption of neutrality, which would indicate a recent population expansion. Tajima’s *D* is widely used in neutrality tests, and Fu’s *F*_S_ and the Ramos–Onsins and Rozas *R*_2_ test were confirmed to be the most powerful tools in examining population growth [43]. The latter was proven to be particularly sensitive for limited sample sizes [43]. Second, population demographic dynamics were investigated through nucleotide mismatch distribution analysis [40,43,44,45] by DnaSP. In addition, the sum of square deviation (SSD) and Harpending’s raggedness index were also calculated by Arlequin. We also calculated the time since population expansion using the equation, *t =* τ*/*2μ, where μ represents the mutation rate of the marker used and τ is a parameter obtained from mismatch distribution analysis in Arlequin. Finally, Bayesian skyline plots were created with BEAST version 1.7.4 [46]. As the mutation rate of D-loop ranged from a minimum 2.2% per million years (myr) in Cichlidae [47] to a maximum of 20% in Clupeidae [48], while that of cyt *b* ranged from 0.68%–2% per myr [49,50], we took 5% per myr [51] as the putative mutation rate for the concatenated sequences. We employed a GTR model with parameters for invariable sites and gamma distribution (GTR + I + G) and ran 10^6^ generations, discarding 10^5^ generations as burn-in. The result was subsequently visualized by Tracer 1.5 [46].

## 5. Conclusions

*C. chinensis* along the Chinese coast displayed the absence of significant population structure and genetic differentiation. In its demographic history, it experienced a low effective population size during the Quaternary period that increased sharply after the last glacial maximum (LGM). The phylogeographic pattern of *C. chinensis* may be attributed to past population expansion and long-distance pelagic larval dispersal facilitated by present-day ocean currents.

## Supplementary Files

Supplementary File 1
