# Supplementary material for: Phylogeography of the Chinese Beard Eel, Cirrhimuraena chinensis Kaup, Inferred from Mitochondrial DNA: A Range Expansion after the Last Glacial Maximum
Source: Int J Mol Sci. 2014 Aug 5;15(8):13564–77. doi: 10.3390/ijms150813564 (PMC4159811; doi:10.3390/ijms150813564)

Supplementary Information

Table S1. Summary of sample size, haplotype numbers, haplotype diversity (*h*), nucleotide diversity (π), Tajima's D, Fu's *F*s, Ramos-Onsins & Rozas R_2 tests and goodness-fit tests for cyt *b* sequences in each population, respectively. * $p < 0.05$; and ** $p < 0.01$.

Populations (Abbreviation)	Sample Size (*n*)	Haplotype Numbers	Haplotype Diversity (*h*)	Nucleotide Diversity (*π*)	Tajima's *D*	Fu's *F*s	Ramos-Onsins & Rozas R_2	SSD	Raggedness Index
**Ningde(ND)**	49	16	0.798	0.002	−1.935 **	−10.989 **	0.022 **	0.001	0.041
**Xiamen(XM)**	37	12	0.581	0.001	−2.357 **	−9.063 **	0.036 **	0.002	0.049
**Shantou(ST)**	30	11	0.605	0.002	−1.958 *	−6.667 **	0.013 **	0.462	0.041
**Yangjiang(YJ)**	50	21	0.731	0.002	−2.482 **	−21.129 **	0.022 **	0.001	0.025
**Beihai(BH)**	35	14	0.717	0.002	−2.167 **	−9.125 **	0.034 **	0.006	0.032
**Haikou(HK)**	37	11	0.641	0.001	−1.750 *	−6.929 **	0.048 **	0.001	0.032
**Sanya(SY)**	28	7	0.582	0.001	−1.808 *	−4.801 **	0.067 **	0.001	0.068
**Total**	266	64	0.700	0.002	−2.569 **	−28.372 **	0.010 **	0.000	0.022

Table S2. Summary of sample size, haplotype numbers, haplotype diversity (*h*), nucleotide diversity (π), Tajima's D, Fu's *F*s, Ramos-Onsins & Rozas R_2 tests and goodness-fit tests for control region (CR) sequences in each population, respectively. * $p < 0.05$; and ** $p < 0.01$.

Populations (Abbreviation)	Sample Size (*n*)	Haplotype Numbers	Haplotype Diversity (*h*)	Nucleotide Diversity (*π*)	Tajima's *D*	Fu's *F*s	Ramos-Onsins & Rozas R_2	SSD	Raggedness Index
**ND**	49	35	0.875	0.001	−2.105 **	−26.599 **	0.035 **	0.008	0.058
**XM**	37	19	0.581	0.001	−1.927 *	−15.622 **	0.026 **	0.001	0.039
**ST**	30	24	0.977	0.003	−1.936 *	−25.882 **	0.053 **	0.009	0.059
**YJ**	50	26	0.900	0.002	−2.317 **	−27.100 **	0.044 **	0.008	0.089
**BH**	35	22	0.943	0.002	−1.460 *	−19.900 **	0.026 **	0.005	0.058
**HK**	37	18	0.887	0.003	−1.853 *	−12.462 **	0.019 **	0.003	0.049
**SY**	28	17	0.851	0.003	−1.880 *	−19.829 **	0.033 **	0.089	0.051
**Total**	266	112	0.916	0.003	−2.340 **	−26.782 **	0.010 **	0.001	0.044

Table S3. Matrix of pairwise of F_{ST} between 7 populations of based on cyt *b* sequences in *Cirrhimuraena chinensis.*

cyt *b*	ND	XM	ST	YJ	BH	HK	SY
**ND**							
**XM**	0.01588						
**ST**	0.00253	−0.00709					
**YJ**	0.00255	−0.00373	−0.01125				
**BH**	−0.00165	−0.0002	−0.01361	−0.00120			
**HK**	0.00950	0.00488	−0.01136	0.00169	−0.01557		
**SY**	0.00639	−0.01606	−0.01085	−0.00621	−0.00249	0.00348	

p values of F_{ST} (not presented here) are all greater than 0.05 and 0.01, which means all the F_{ST} values in the table above are not statistically significant under $p < 0.05$ and $p < 0.01$).

Table S4. Matrix of pairwise of F_{ST} between 7 populations of based on CR sequences in *Cirrhimuraena chinensis.*

CR	ND	XM	ST	YJ	BH	HK	SY
**ND**							
**XM**	−0.00566						
**ST**	−0.00126	−0.00563					
**YJ**	0.00349	−0.00151	0.00339				
**BH**	−0.00752	−0.00834	−0.01477	0.01656			
**HK**	0.00455	0.00390	−0.00007	0.00772	0.01090		
**SY**	0.00234	0.00775	−0.00075	0.00614	0.01712	0.00239	

p values of F_{ST} (not presented here) are all greater than 0.05 and 0.01, which means all the F_{ST} values in the table above are not statistically significant under $p < 0.05$ and $p < 0.01$).

Table S5. AMOVA results for testing genetic subdivision between populations of using cyt *b* fragment among geographic district.

**cyt *b***	**Sum of Squares**	**Percentage of Variation**	**Fixation Indices**	**Significance Tests**
Groups: Taiwan strait (ND)(XM, ST, YJ, BH, HK, SY)				
Among groups	2.178	0.18	$\Phi_{CT} = 0.00182$	$p = 0.571$
Among populations within groups	9.447	0.11	$\Phi_{SC} = 0.00115$	$p = 0.235$
Within populations	469.925	99.70	$\Phi_{ST} = 0.00297$	$p = 0.271$
Groups: Pearl River (ND, XM, ST) (YJ, BH, HK, SY)				
Among groups	1.438	−0.26	$\Phi_{CT} = -0.00283$	$p = 0.917$
Among populations within groups	10.187	0.33	$\Phi_{SC} = 0.00324$	$p = 0.161$
Within populations	469.925	99.93	$\Phi_{ST} = 0.00042$	$p = 0.236$
Groups: Leizhou Peninsula (ND, XM, ST, YJ, HK, SY) (BH)				
Among groups	2.010	0.09	$\Phi_{CT} = -0.00259$	$p = 0.696$
Among populations within groups	9.614	0.16	$\Phi_{SC} = 0.00328$	$p = 0.220$
Within populations	469.925	99.76	$\Phi_{ST} = 0.00070$	$p = 0.242$
Groups: Qiongzhou Strait (ND, XM, ST, YJ, BH) (HK, SY)				
Among groups	2.170	0.16	$\Phi_{CT} = 0.00159$	$p = 0.288$
Among populations within groups	9.454	0.11	$\Phi_{SC} = 0.00110$	$p = 0.359$
Within populations	469.925	99.73	$\Phi_{ST} = 0.00269$	$p = 0.281$

Table S6. AMOVA results for testing genetic subdivision between populations of using CR fragment among geographic district.

CR	Sum of Squares	Percentage of Variation	Fixation Indices	Significance Tests
Groups: Taiwan strait (ND)(XM,ST,YJ,BH,HK,SY)				
Among groups	2.178	0.18	$\Phi_{CT} = 0.00182$	$p = 0.573$
Among populations within groups	9.447	0.11	$\Phi_{SC} = 0.00115$	$p = 0.275$
Within populations	469.925	99.70	$\Phi_{ST} = 0.00297$	$p = 0.269$
Groups: Pearl River (ND,XM,ST)(YJ,BH,HK,SY)				
Among groups	1.463	−0.28	$\Phi_{CT} = -0.00283$	$p = 0.897$
Among populations within groups	10.161	0.33	$\Phi_{SC} = 0.00324$	$p = 0.152$
Within populations	469.925	99.96	$\Phi_{ST} = 0.00042$	$p = 0.284$
Groups: Leizhou Peninsula (ND,XM,ST,YJ,HK,SY)(BH)				
Among groups	2.010	0.09	$\Phi_{CT} = 0.00085$	$p = 0.710$
Among populations within groups	9.614	0.16	$\Phi_{SC} = 0.00156$	$p = 0.229$
Within populations	469.925	99.76	$\Phi_{ST} = 0.00242$	$p = 0.271$
Groups: Qiongzhou Strait (ND,XM,ST,YJ,BH)(HK,SY)				
Among groups	2.010	0.16	$\Phi_{CT} = 0.00085$	$p = 0.713$
Among populations within groups	9.614	0.09	$\Phi_{SC} = 0.00156$	$p = 0.270$
Within populations	469.925	99.76	$\Phi_{ST} = 0.00242$	$p = 0.260$

Figure S1. Median-joining network of *Cirrhimuraena chinensis* Kaup for concatenated sequences (cyt *b* + CR) using software NETWORK.

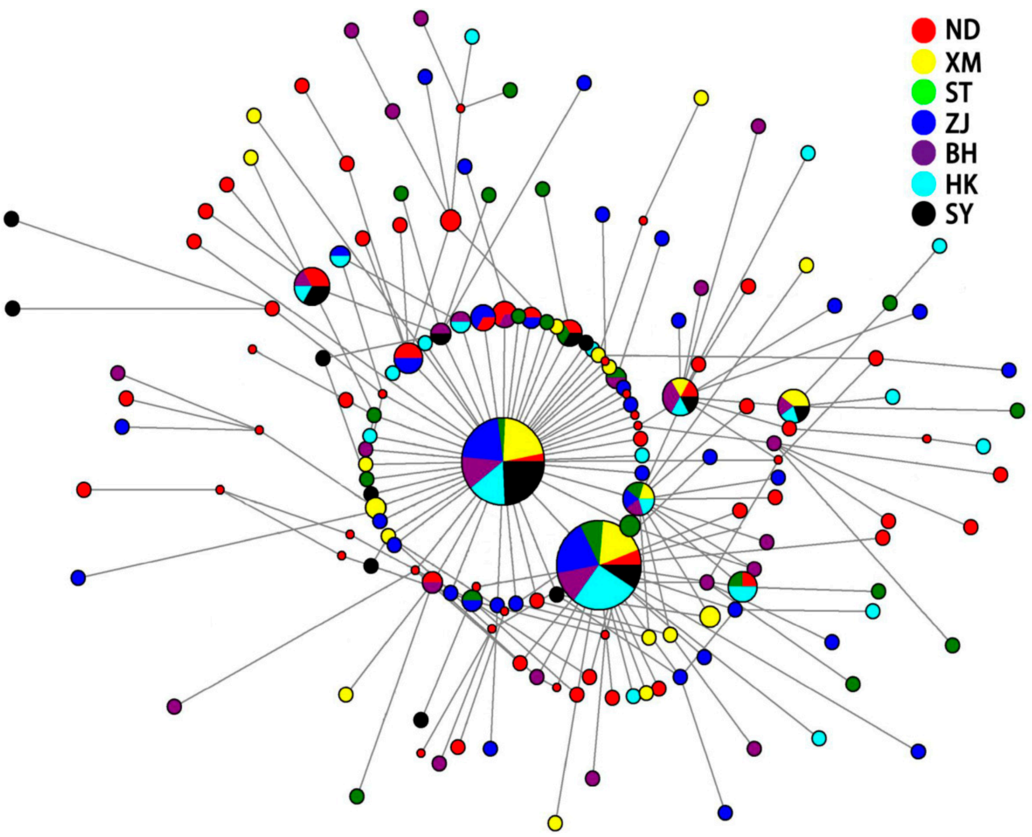

Figure S2. Median-joining network of *Cirrhimuraena chinensis* Kaup for concatenated sequences (cyt *b* + CR) using software TCS.

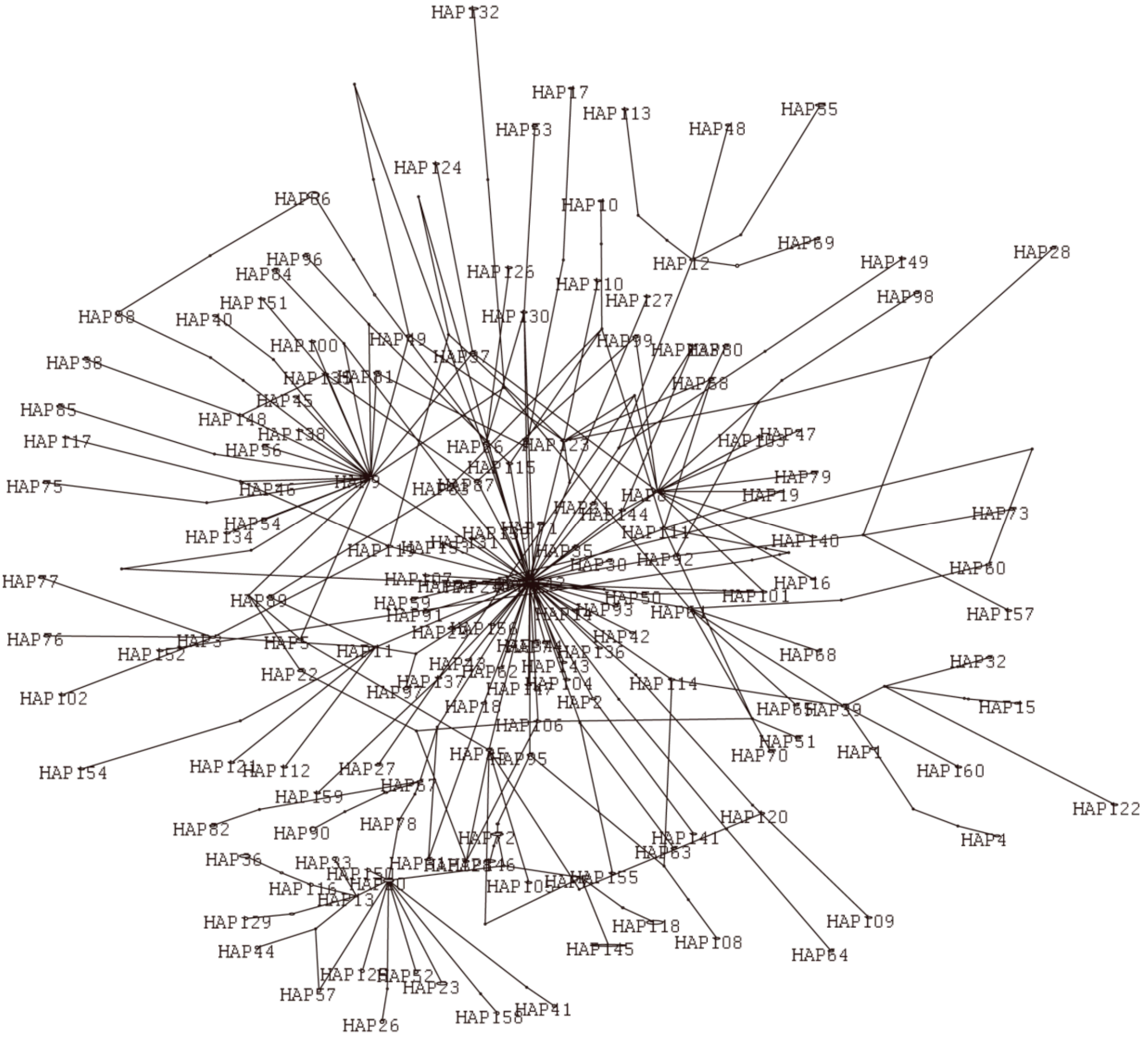

Figure S3. Bayesian Skyline Plots of the effective sizes through time for *Cirrhimuraena chinensis* Kaup based on cyt *b* fragment. The upper and lower limits of light blue trend represent the 95% confidence intervals of highest posterior densities (HPD) analysis.

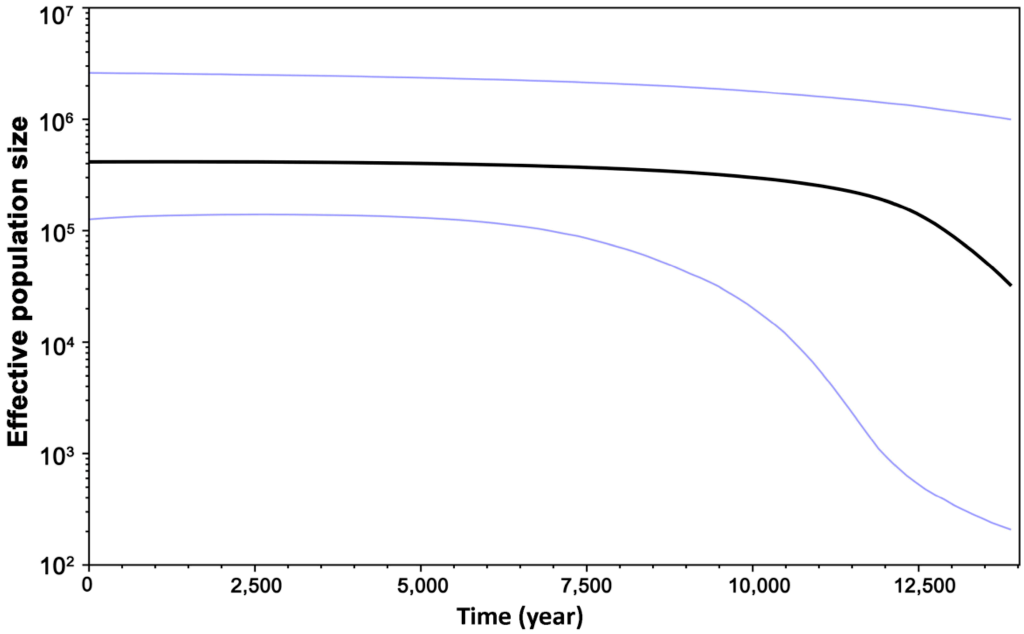

Figure S4. Bayesian Skyline Plots of the effective sizes through time for *Cirrhimuraena chinensis* Kaup based on cyt *b* fragment. The upper and lower limits of light blue trend represent the 95% confidence intervals of HPD analysis.

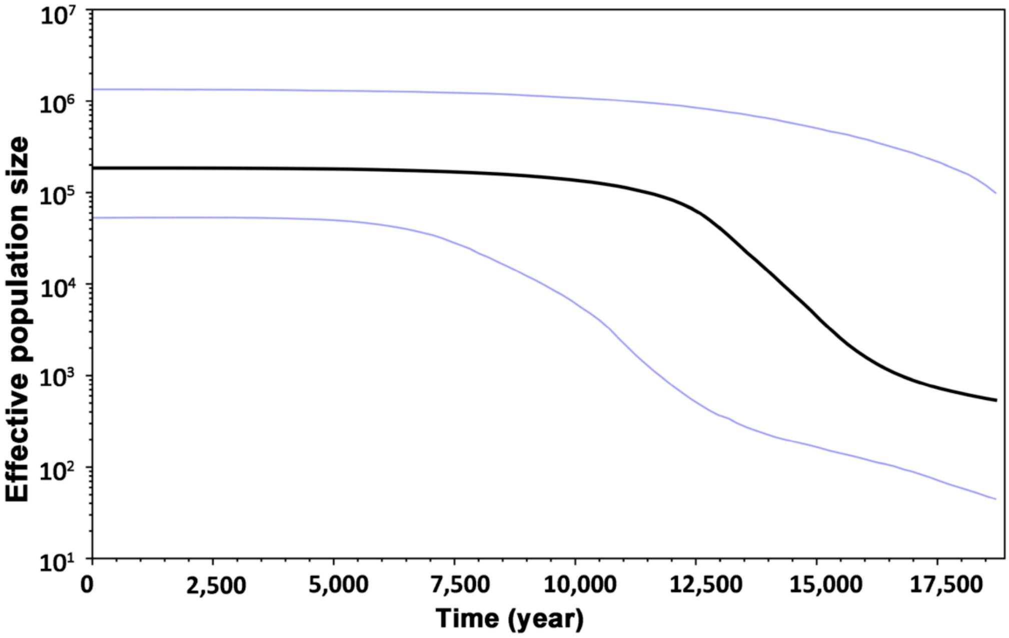

Supplement: Supplementary File 1 [file ijms-15-13564-s001.pdf]
